# Monitoring training status with HR measures: do all roads lead to Rome?

**DOI:** 10.3389/fphys.2014.00073

**Published:** 2014-02-27

**Authors:** Martin Buchheit

**Affiliations:** Sport Science Department, Myorobie AssociationMontvalezan, France

**Keywords:** heart rate variability, heart rate recovery, training response, fatigue, endurance sports, team sports, assessing changes, progressive statistics

## Abstract

Measures of resting, exercise, and recovery heart rate are receiving increasing interest for monitoring fatigue, fitness and endurance performance responses, which has direct implications for adjusting training load (1) daily during specific training blocks and (2) throughout the competitive season. However, these measures are still not widely implemented to monitor athletes' responses to training load, probably because of apparent contradictory findings in the literature. In this review I contend that most of the contradictory findings are related to methodological inconsistencies and/or misinterpretation of the data rather than to limitations of heart rate measures to accurately inform on training status. I also provide evidence that measures derived from 5-min (almost daily) recordings of resting (indices capturing beat-to-beat changes in heart rate, reflecting cardiac parasympathetic activity) and submaximal exercise (30- to 60-s average) heart rate are likely the most useful monitoring tools. For appropriate interpretation at the individual level, changes in a given measure should be interpreted by taking into account the error of measurement and the smallest important change of the measure, as well as the training context (training phase, load, and intensity distribution). The decision to use a given measure should be based upon the level of information that is required by the athlete, the marker's sensitivity to changes in training status and the practical constrains required for the measurements. However, measures of heart rate cannot inform on all aspects of wellness, fatigue, and performance, so their use in combination with daily training logs, psychometric questionnaires and non-invasive, cost-effective performance tests such as a countermovement jump may offer a complete solution to monitor training status in athletes participating in aerobic-oriented sports.

## Introduction

Training responses in athletes are generally related to the training stimuli (e.g., relative/internal training load) during the different training cycles (Borresen and Lambert, 2009). While too much and too little training load may lead to accumulated fatigue (non-functional overreaching or overtraining) and detraining, respectively, an appropriate training dose at the individual level may allow optimal improvements in fitness and performance (Bouchard and Rankinen, 2001; Hautala et al., 2006, 2009; Borresen and Lambert, 2009; Manzi et al., 2009b, 2013; Castagna et al., 2011). It is therefore paramount to monitor athletes' fatigue, fitness and/or performance responses to the various training phases, so that training load and contents can be adjusted and individualized both during and between each training cycles (Borresen and Lambert, 2009; Kiely, 2012; Plews et al., 2013b; Stanley et al., 2013a). The quantification of training load is generally based on both external (distance, power output, number of repetitions) and internal (oxygen uptake, heart rate, blood lactate, rate of perceived exertion) indicators of effort intensity, which are then computed with training time to derive compound training load measures (e.g., training stress score using power output Skiba, 2006, training impulse using HR Banister and Hamilton, 1985, perceived load using session rate of perceived exertion, RPE Foster, 1998). When it comes to monitoring athletes' fatigue and/or performance responses to their training load, there are today various tools and methods, such as the monitoring of saliva and specific blood variables (Heisterberg et al., 2013; Meeusen et al., 2013), or the use of psychometric questionnaires (Hooper and Mackinnon, 1995; Borresen and Lambert, 2009; Buchheit et al., 2013a). Changes in blood lactate might be used to track changes in fitness (Beneke et al., 2011), but whether they can inform on fatigue *per se* is still debated (Le Meur et al., 2013a; Meeusen et al., 2013), especially during submaximal exercise. Moreover, blood samples may be painful and are inconvenient for frequent monitoring. There is therefore an increasing interest in monitoring the status of the autonomic nervous system (ANS) via measures of heart rate (HR), including the level and variability of HR at rest (Plews et al., 2013b; Stanley et al., 2013a) and following exercise (Buchheit et al., 2008, 2010a, 2012, 2013a), during exercise (Rowsell et al., 2009; Buchheit et al., 2010a, 2012; Garvican et al., 2013) and during recovery after exercise (Daanen et al., 2012).

The main interest of HR measures is that they are non-invasive, not expensive, time-efficient and can be applied routinely and simultaneously in a large number of athletes. While the collection of (beat-by-beat) HR was initially only possible with expensive laboratory-based electrocardiograph recorders, the recent availability of valid and portable recorders such as heart-rate monitors (Gamelin et al., 2006; Weippert et al., 2010; Wallen et al., 2012), specifically-designed systems (Parrado et al., 2010; Cassirame et al., 2013) or smart phone applications (Flatt and Esco, 2013) has substantially boosted the use of HRV monitoring in the field. However, despite its common implementation in the field, HR monitoring is still not accepted as a gold standard, likely due to the lack of consistency in the literature. While some studies have shown that these measures are sensitive to fitness improvements, fatigue, overload or detraining, others have not (Plews et al., 2013b). Changes opposite to those expected have also been observed, which further complicates the interpretation of athletes' training status (Plews et al., 2013b). Based on our personal experiences and some collaborative work with elite athletes in the field and in the laboratory, we contend that most of the contradictory findings published to date may be related to methodological inconsistencies and/or partial misinterpretation of the data rather than to limitations of HR measures to accurately inform on training status (Plews et al., 2013b).

In this technology report, I provide evidence that (at least some of) these HR measures can be successfully used to inform on (1) acute fatigue/recovery responses to isolated aerobic-oriented training sessions, and, in turn, adjust training load on a day-to-day basis, and (2) inform on both positive and negative adaptations to aerobic-oriented training blocks. To do so, the possible applications of each measure, together with the methodological issues that need to be considered to use those measures confidently will be discussed. Statistical guidelines will be presented to correctly interpret HR indices changes with training. Finally, a simple decision process will be offered to assist practitioners in selecting the HR variables most suited to their needs.

## One simple variable, multiple complex indices

While measuring pulse rate is handy and straightforward, it can be measured at different occasions (i.e., during sleep or awake at rest, during or following exercise), and its analysis can be as simple as complex (e.g., HR values vs. HR variability, HRV). All recording conditions and methods of analysis have advantages and disadvantages, which all need to be understood when selecting the most appropriate monitoring methodology with athletes. The physiological determinants of the different indices differ (Buchheit et al., 2007), and their time course of adaptation during training is likely different (Buchheit et al., 2010a). Their sensitivity to fitness, overload and performance is also likely different (Buchheit et al., 2010a, 2012, 2013a). For example, in the only training study in (recreational) athletes where most of these HR-derived indices were measured simultaneously, improvements in exercise HR (HRex) and post-exercise HR recovery (HRR) occurred within a week after the start of an endurance training program (Buchheit et al., 2010a). Conversely, changes in resting HRV were observed only after the 4th training week (Buchheit et al., 2010a). The associations with fitness and running performance were also HR measure–dependent. Compared with HRex, vagal-related HRV indices might be a better predictor of, or might share more common determinants with prolonged (e.g., 10-km time) than short (e.g., incremental tests) aerobic-related performance. HRR is generally accelerated in athletes with high training volumes (Darr et al., 1988; Buchheit and Gindre, 2006), and both HRV and HRR respond well to acute changes in training load (i.e., the greater the training load, the smaller the vagal-related HRV Pichot et al., 2000, 2002 and the slower the HRR Borresen and Lambert, 2007). While all these measures are somehow related to the ANS activity, these results show that each variable might inform on a different physiological aspect of adaptation (Buchheit and Gindre, 2006; Buchheit et al., 2007, 2010a). Their use in combination might therefore improve the monitoring of athletes. Although the appropriate combination, timing, and methodology of data collection still need to be determined, there is evidence demonstrating that a regular monitoring of a few of those variables can substantially improve the training process (Buchheit et al., 2010a; Daanen et al., 2012; Plews et al., 2013b). In the following section I will start by presenting the physiological determinants and the practical usefulness of each measure separately.

### Resting measures

**Figure 1:**
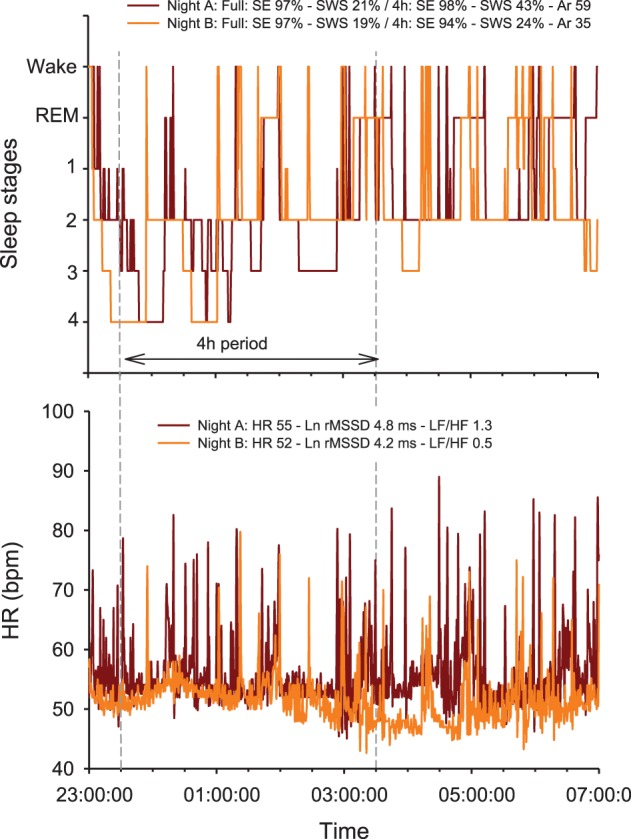
**Overnight hypnogram and heart rate (HR) patterns during two nights subjectively rated as very good (i.e., 5/5 on a 5-point scale) by a 25-year old team sport athlete.** Physical activity (no intense exercise) preceding each night was similar (resting day, i.e., 2809 vs. 2826 Kcal, as measured by Tri-axial accelerometers) (Buchheit et al., 2004; Brandenberger et al., 2005). Note that while both sleep efficiency (SE) and total slow wave sleep (SWS) time are similar over the two complete nights (which is consistent with the subjective rating of the night), the actual sleep stage distribution and fragmentation between the first 4 h of the 2 nights differ markedly [i.e., greater proportion of SWS during the first part of Night A, 43 vs. 24%, and more arousals (Ar, changes in sleep stages), 59 vs. 35]. The greater sleep fragmentation during night A is associated with more HR arousals, which directly increases (1) the number of rapid (beat-by-beat) changes in HR [reflected by both the power density in the high frequencies (HF) or the square root of the mean of the sum of the squares of differences between adjacent normal R-R intervals, Ln rMSSD] and (2) more importantly, the power spectral density in the low frequencies (LF), which, in turn, increases dramatically the LF/HF ratio. Compared with Night B, this results in a paradoxical autonomic response, where a greater vagal activity (Ln rMSSD) is super-imposed to a greater sympathetic background (greater HR and LF/HF ratio). Autonomic co-activation may explain these observations (Tulppo et al., 1998a), however, this is more likely a limitation of the HRV analysis methods to capture the actual ANS state during non-stationary periods such as sleep. Additionally, the greater Ln rMSSD and LF/HF values are inconsistent with the greater proportion of SWS during the first part of Night A, since SWS is generally is generally associated with both reduced Ln rMSSD and LF/HF (Otzenberger et al., 1998; Brandenberger et al., 2005). Therefore, the calculation and interpretation of HRV indices over a large period of sleep, with no consideration of the actual sleep stage patterns, is particularly challenging and remains questionable for monitoring purposes. REM: rapid eye movements.

The physiological determinants of resting HR are multiple and include cardiac muscle morphology, plasma volume, autonomic activity, age and body position; resting HRV is essentially related to genetics, plasma volume, autonomic activity and body position (Table 1) (Achten and Jeukendrup, 2003; Aubert et al., 2003; Sandercock and Brodie, 2006; Bosquet et al., 2008b). The basic principle of HR(V) monitoring is to make inferences on possible changes in cardiac ANS status with training, while using repeated HR(V) measures over the time. Since ANS activity is highly sensitive to environmental conditions (e.g., noise, light, temperature Achten and Jeukendrup, 2003), it is important that precautions be taken to standardize recording conditions in order to isolate the training-induced effects on ANS. Therefore, night recordings represent theoretically the best (more standardized) recording condition for HR(V) monitoring (Pichot et al., 2000; Brandenberger et al., 2005). However, the effects that differences in sleep patterns (Otzenberger et al., 1998) and quality (Burton et al., 2010) have on HRV, independent of training-related changes in ANS status, have been overlooked in the literature, and consequently lead to misinterpretation (Figure 1, see text legend for further discussion). To overcome these limitations, HRV data collected during select slow wave sleep episodes, that offers great signal stability and a high standardization of both environmental factors and respiratory influences on HRV, might be preferred (Buchheit et al., 2004; Brandenberger et al., 2005). Interestingly, because of the particular shape of the Poincaré plot during this sleep stage (i.e., narrow round shape Otzenberger et al., 1998), slow wave sleep-related HRV measures can be selected without the need of an hypnogram (Vinet et al., 2005; Stanley et al., 2013b). However, in practice with athletes, night recordings are noisy and difficult to implement daily (Stanley et al., 2013b), limiting their usefulness. Furthermore, the level of activity the previous day tends to affect night HRV during the early hours of sleep, where the greater proportion of SWS occurs (Myllymaki et al., 2011). As it will be detailed in section Methodological Considerations, consideration of training load from the day preceding the measure is crucial to correctly interpret the changes in ANS status from HRV recordings.

**Table 1:** **Determinants, reliability and sensitivity of the different heart rate measures**.

	**Determinants**	**Monitoring variable(s)**	**Typical error, expressed as coefficient of variation**	Signal-to-noise ratio^*^	Smallest worthwhile change^*^
Resting HR	Cardiac morphology, plasma volume, ANS and baroreflex	Wellness, fitness, readiness to perform	~10%	0.7	~ −2%
Resting vagal-related HRV indices	Genetics, plasma volume, ANS and baroreflex	Wellness, fitness, readiness to perform	Index-dependent, e.g., ~12 (Ln rMSSD) to ~80 (LF/HF)%	0.8	~ +3%
Exercise HR	Fitness, plasma volume	Aerobic fitness	~3%	1.6	~ −1%
Exercise HRV	Intensity-dependent: ANS<VT1, respiration >VT2	In theory, aerobic fitness	Index-dependent, e.g., ~60 (Ln rMSSD) to ~150 (LF/HF)%	N/A	N/A
Post-exercise HRR	Theoretically ANS and genetics but essentially metaboreflex	In theory, wellness, fitness and readiness to perform In practice, more fitness because of its link with relative exercise intensity	Index-dependent, e.g., ~25 (HRR_60s_) to ~35 (HRRτ)%	1.3	~ +7%
Post-exercise vagal-related HRV indices	ANS and baroreflex, but the metaboreflex has the greater effect	In theory, wellness, fitness and readiness to perform In practice, more fitness because of its link with relative exercise intensity	Index-dependent, e.g., ~16 (Ln rMSSD) to ~65 (LF/HF)%	1.1	~ +4%

HR, heart rate; HRR_60 s_, HR recovery within 60 s following exercise; HRRτ, time constant of HR recovery derived from monoexponential modeling; HRV, HR variability; N/A, non-available. Typical error: see Acknowledging the Uncertainty of the Measures text for related references. Signal to noise ratio: refers to the magnitude of the change observed in Buchheit et al. (2010a) divided by the typical error reported in Al Haddad et al. (2011). See section Determining the Smallest Worthwhile Change text for the methods used to calculate the smallest worthwhile change.

* , for positive training adaptations.

**Figure 2:**
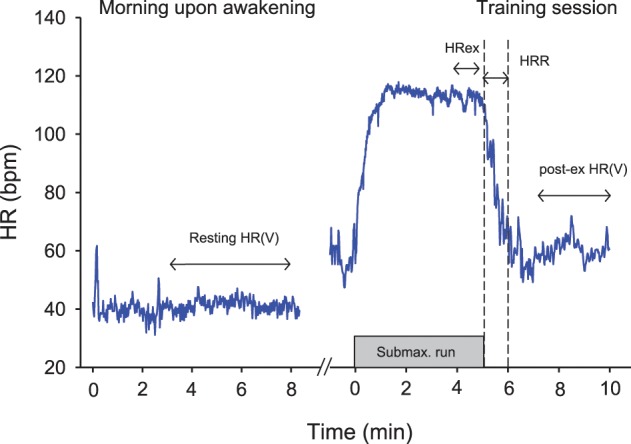
**Example of the different heart rate (HR) recording conditions during the day time.** HRex, exercise HR; HRR, HR recovery over 60s; HRV, HR variability.

Currently, the best practice for athletes tends to be short-term (5–10 min) measurements of HR(V) upon awakening in the morning (Figure 2) (Plews et al., 2013b; Stanley et al., 2013a). While both supine and standing recordings are often used in the literature (Schmitt et al., 2013), the supine condition is better tolerated by athletes in the field. Seated recording are also of great interest for athletes' comfort, and there are definitely less chances for the athletes to fall asleep than during supping measures. Whether the more constraining standing recordings reveal extra information compared with supine is unclear at the moment. Such morning resting measures allow overcoming the limitations of night recordings, while still offering a kind of standardization (e.g., same bed and same time most days, quiet environment, no immediate effect of daily activities). Research comparing night vs. morning measures is limited and has shown conflicting results (Buchheit et al., 2004; Hynynen et al., 2006). It is also surprising that while HR has been used for decades by coaches, scientists have essentially focused their researches on HRV, considering it to be a more powerful tool. However, the possibly greater sensitivity of HRV to training status is unlikely as large as previously thought (see section Interpreting Changes in Heart Rate Measures: “Statistics are our weapons”). In a recent study, there was only a slightly stronger trend toward non-functional overreaching for vagal-related HRV indices compared with HR (*r* = 0.88 vs. 0.81) (Plews et al., 2012). Similarly, the correlations between changes in resting HRV and 10-km running performance (*r* = 0.76) were only slightly greater than when using changes in HR (*r* = 0.73) (Plews et al., 2013a). Further research with larger sample sizes and examination of different training status are still required to draw definitive conclusions.

There is a plethora of methods to assess HRV (Task Force, 1996). The most commonly used methods are time domain and spectral analyses; non-linear methods such as entropy and symbolic analyses have also gained some interest more recently (Task Force, 1996). Each index captures a different feature of the ANS, with some indices more likely to reflect cardiac sympathetic activity, while others, cardiac parasympathetic activity (Task Force, 1996). In practice however, when it comes to selecting the more appropriate HRV indices to monitor athletes in the field, time domain indices [e.g., rMSSD (square root of the mean of the sum of the squares of differences between adjacent normal R-R intervals) or SD1 (standard deviation of instantaneous beat-to-beat R–R interval variability measured from Poincaré plots), both reflecting parasympathetic modulation] are the most attractive ones. First, these indices can be captured over a very limited period of time (e.g., 10 s to 1 min Billman and Hoskins, 1989; Hamilton et al., 2004; Nussinovitch et al., 2011a), and are compatible with the short duration of the recordings usually performed by athletes on the field. Second, compared with spectral indices, the sensitivity of rMSSD and SD1 to breathing patterns is very low (Penttila et al., 2001), which is also well suited for day-to-day monitoring under spontaneous breathing in athletes (Saboul et al., 2013). Third, since rMSSD can be calculated with an Excel spreadsheet, it doesn't require any sophisticated software package and is therefore accessible to anyone. Fourth, the day-to-day variations of time domain indices during short (5–10 min) recordings in the field is likely lower than these of spectral indices, especially when considering the ratios (e.g., coefficient of variation (CV) for rMSSD ≈12% vs. low-to-high frequency oscillations ratio, LF/HF, CV ≈82% Al Haddad et al., 2011). While the absolute magnitude of a CV is not an issue *per se* (see section Interpreting Changes in Heart Rate Measures: “Statistics are our weapons”), large CVs may decrease the signal-to-noise ratio, which could compromise the sensitivity of the measures. To conclude, in contrast to previous recommendations for short-term recordings under laboratory settings (Task Force, 1996), I discourage practitioners using spectral indices in the field. I contend that for the reasons mentioned above, resting HR and time domain HRV indices such as rMSSD should be collected in priority at rest in athletes. Despite large discrepancies in the literature (probably related to methodological issues, see sections Methodological Considerations and Interpreting Changes in Heart Rate Measures: “Statistics are our weapons”), these indices, when interpreted correctly, are two promising tools to monitor changes in general fatigue, fitness and performance both over short (Stanley et al., 2013a) and longer training periods (Plews et al., 2013b).

### Exercise measures

#### Exercise HR

Together with resting HR, HRex (Figure 2) is probably the easiest measure to collect, and has the advantage that it does not require advanced HR monitors (i.e., beat-by-beat measures are not needed). Depending on the exercise intensity, at least 3–4 min of exercise are generally required for HR to reach a steady state during submaximal exercise (Cerretelli and Di Prampero, 1971). The average HR over the last 30–60 s is generally used for analysis (e.g., Buchheit et al., 2008, 2010a). Since HR is closely related to O_2_ uptake during continuous exercise, HRex (when expressed as a percentage of maximal HR) provides a good marker of within-athlete relative exercise intensity, with the lower the HR, the fitter the athletes (Mann et al., 2013). For instance, there are large to very-large correlations between decreases in HRex and improvements in high-intensity exercise performance (Bangsbo et al., 2008; Buchheit et al., 2008, 2010a, 2011b, 2012, 2013a,b; Lamberts et al., 2010, 2011b; Lamberts, 2013). Importantly however, although this has been claimed by some authors (Brink et al., 2010, 2013), an increased HR should not be used as a clear marker of fatigue and/or fitness impairment (Buchheit et al., 2012). Recent data suggest also than in the particular context of high-altitude camps, a >4% increase in HRex in response to an increased training load the day before could be predictive of sickness the following day (Buchheit et al., 2013c). In summary, HRex is a promising tool to monitor positive aerobic-oriented training adaptations (Buchheit et al., 2012), with a greater ability to predict changes within the first months of a training program (Scharhag-Rosenberger et al., 2009). Whether HRex can be confidently used to predict sickness requires confirmation in larger populations and under other environmental conditions.

#### Heart rate variability during exercise

HRV measures during exercise have been used as a measure of fitness level (Tulppo et al., 1996, 1998b; Sandercock and Brodie, 2006; Lewis et al., 2007). However, limited data exists in humans on their ability to track training-induced changes in fatigue and/or fitness within the same individuals (Billman and Hoskins, 1989; Kukielka et al., 2006). Indeed, there are several limitations to their usefulness as a training monitoring tool. First, the determinants of HRV during exercise are intensity-dependent, and non-exclusively ANS-related (Blain et al., 2005; Sandercock and Brodie, 2006; Buchheit and Mendez-Villanueva, 2010). While below the first ventilatory threshold (VT1), vagal activity likely contributes to the greater proportion of HRV, respiratory fluctuations determine HRV at greater intensities, especially after the respiratory compensation point (Blain et al., 2005). Therefore, to ensure that it is the ANS that is captured through HRV, exercise intensity has to be tightly individualized (i.e., ≤ individual VT1) (Buchheit et al., 2007). However, this is unpractical in the field when testing several team sport athletes at a time, where players run generally at a similar speed (Buchheit et al., 2012, 2013a). Additionally, during exercise, beat-to-beat recordings are generally noisy (e.g., erroneous or lost beats due to movements of the HR monitor belt). While this is not an issue when averaging beats over 30–60 s (HRex), this requires extensive data treatment/cleaning and sometime prevents the proper calculation of HRV indices. In conclusion, until new evidence is presented, HRV during exercise appears to be more of a scientific toy than a real training monitoring tool.

### Post-exercise measures

#### Post-exercise heart rate recovery

Post exercise HRR reflects general hemodynamic adjustments in relation to body position, blood pressure regulation and metaboreflex activity, which partly drives sympathetic withdrawal and parasympathetic reactivation (Buchheit et al., 2007; Daanen et al., 2012). In highly-trained young soccer players, no clear association between HRR and blood pressure variability (expected to reflect sympathetic vasomotor tone) was observed (Buchheit et al., 2011a). The dissociation between post-exercise changes in HR and stroke volume is also important, suggesting that HRR does not correctly reflect the time course of cardiac output recovery (Takahashi and Miyamoto, 1998; Buchheit et al., 2011a). A wide range of methodologies are available to measure HRR, from the number of beats recovered within a given time (e.g., 60 s, HRR_60 s_) to signal modeling via linear or (mono)exponential models (Buchheit et al., 2007). While direct comparisons of the sensitivity to training of all HRR variables are scarce, monoexponential modeling may better capture the overall HR response (with both the initial fast component and the delayed recovery phase being taken into account). Conversely, signal modeling requires sophisticated software and is time-consuming, which may limit its application with large numbers of athletes involved in regular monitoring. In practice, correlations between changes in simple HRR variables (i.e., HRR_60s_) and both changes in endurance performance and fatigue have been reported (Daanen et al., 2012). It has been suggested that HRR is a relevant training monitoring tool to track positive changes in high-intensity exercise performance. However, in team sports especially, changes in HRR did not always correlate with performance changes (Buchheit et al., 2012, 2013a), or displayed correlations of lower magnitude than that observed with HRex. Additionally, whether HRR can also track impairment in performance (Mann et al., 2013), as does resting HRV (Plews et al., 2012), has to be confirmed in a wider range of athletes and sports.

#### Post-exercise heart rate variability

Post-exercise HRV has received a growing interest within the past decade, with the belief that it would bring better information on training adaptations than that provided by resting HRV or HRR (Yamamoto et al., 2001). The determinants of post-exercise HRV are multiple, and include blood pressure regulation, baroreflex activity, and especially metaborelfex stimulation following exercise, that drives sympathetic withdrawal and parasympathetic reactivation (Buchheit et al., 2007; Stanley et al., 2013a). The greater the relative exercise intensity, the greater the blood acidosis and metaboreflex stimulation, and the slower the HRR and the lower the vagal-related HRV indices. To assess the “true” autonomic influences on HR rhythm independent of the metaborelfex stimulation, the use of submaximal exercise only (≤VT1) has been suggested (Buchheit et al., 2007). If the intensity is not low enough, post-exercise HRV is very largely related to exercise intensity, and it becomes redundant with HRex (in addition to be less reliable, Table 1). Further, in team sports it is difficult to have all players running at a similar relative intensity in the field. To conclude, despite some promising results (Yamamoto et al., 2001), post-exercise HRV might actually have a similar (Buchheit et al., 2010a) or even lower (Buchheit et al., 2012, 2013a) usefulness than HRex and morning resting HRV (Buchheit et al., 2012, 2013a). This is likely related to the fact that while HRex and morning resting HRV have clearly distinct determinants (i.e., relative exercise intensity for HRex and cardiac ANS activity for resting HRV), post-exercise HRV is a compound measure influenced by (too) many factors.

## Methodological considerations

### R-R series editing

**Figure 3:**
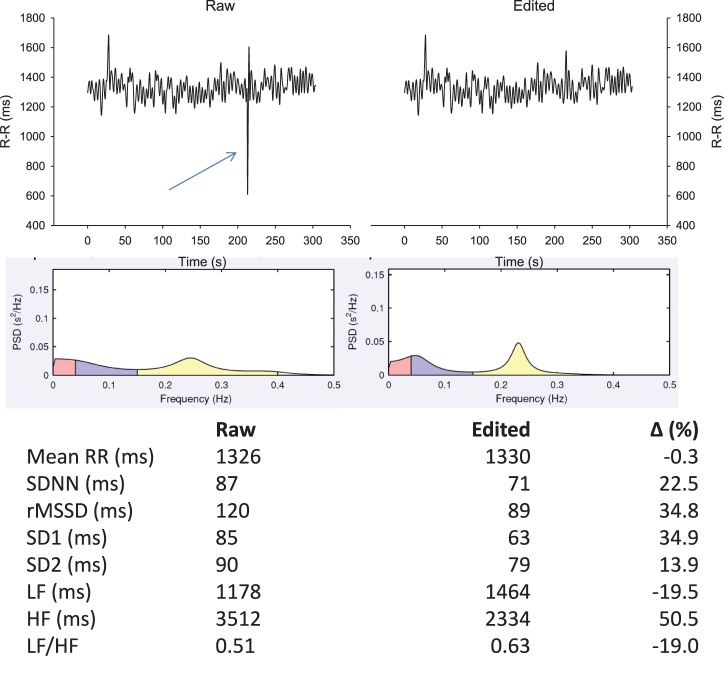
**Effect of an ectopic beat on traditional heart rate (HR) variability (HRV) indices.** R-R intervals recorded in supine position for 5 min with data either non-edited (upper left panel) or with beat removal and linear interpolation with adjacent values (upper right panel). Middle panels show the associated power spectral density (PSD) distribution on a spectrogram (Kubio HRV, 2.1, Biosignal Analysis and Medical Imaging Group, Kuopio, Finland) and lower panels, the common HRV indices derived from those two R-R series. SDNN, standard deviation of normal R-R intervals; rMSSD, square root of the mean of the sum of the squares of differences between adjacent normal R-R intervals; SD1, standard deviation of instantaneous beat-to-beat R-R interval variability measured from Poincaré plots; SD2, standard deviation of long-term beat-to-beat R-R interval variability measured from Poincaré plots; LF, low-frequency oscillations, HF, high-frequency oscillations.

The importance of R-R series editing prior to analyse is often overlooked (Task Force, 1996). As shown in Figure 3, the presence of a single ectopic beat (or a skipped beat) over a 5-min recording can modify common HRV indices up to 50%. Since these differences might not reflect real changes in the ANS status, a proper editing of R-R series before analysis is crucial. While the examination of the complete ECG trace is required to draw definitive conclusions on the nature of “abnormal” heart beats (i.e., missed vs. ectopic beats), doing so is unrealistic in practice when dealing with a large number of files daily. R-R series are often automatically edited within manufacturers' software (beat removal and linear extrapolation from adjacent beats) (Nunan et al., 2009). While a few errors may still occur using these automatic filters (e.g., removal of ANS-generated change in HR), this is not a major issue for athlete monitoring when their use is consistent prior to each analysis. The impact of ectopic beats on HRR are likely similar than for HRV when fitting a monoexponential model, since those beats can distort the overall fit and in turn, the time constant of the exponential. Ectopic beats are likely less problematic for HRR_60 s_, since the beats used for calculation are generally averaged over a few seconds (Buchheit et al., 2007).

### Responses to training sessions, training intensity distribution and environmental conditions

#### Acute responses

**Figure 4:**
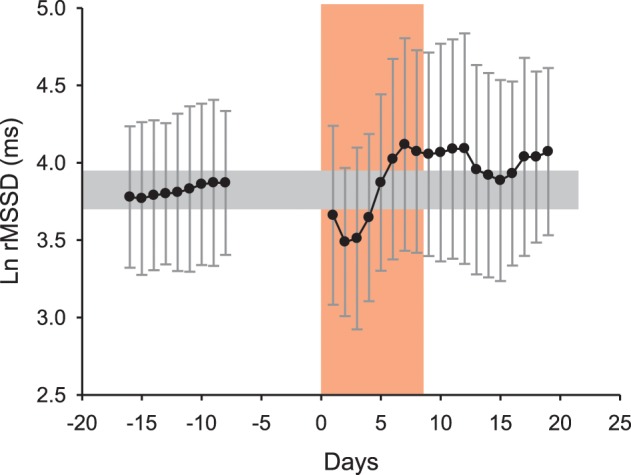
**Average changes (90% confidence intervals) in the logarithm of the square root of the mean of the sum of the squares of differences between adjacent normal R–R intervals (Ln rMSSD) measured at rest after awakening in 6 runners (4M-2F, 38.2 ± 1.8 years, 170.7 ± 7.9 cm, 65.0 ± 11.6 kg, 6.8 ± 2.1 h of training per week) before, during and following the 20th “Marathon des sables” (2005, 253 km in 7 days under hot environment, with temperature sometimes exceeding 50°C).** The increase in Ln rMSSD after the 4th day of race is unlikely reflective of general fatigue and fitness (which were actually reported to be deteriorated by the runners) but rather reflects changes in plasma volume consecutive to the combination of exercise-induced hypervolemia (Buchheit et al., 2009) and heat acclimatization responses (Ladell, 1951; Buchheit et al., 2013a). Unpublished data (Buchheit et al.).

The day-to-day variations in training load entail large daily variations in cardiac ANS activity (i.e., *CV* = 10–20% for Ln rMSSD Buchheit et al., 2010b, 2013a). In general, intense exercise acutely decreases vagal-related HRV indices for 24–48 h, that may coincide with homeostasis restoration and perceived levels of overall recovery (Stanley et al., 2013a). Following these observations, these day-to-day variations in HRV have been used to guide training contents on a daily basis (i.e., train at high intensity only when vagal-related HRV indices have returned back to normal levels) (Kiviniemi et al., 2007, 2010; Stanley et al., 2013a). Such a HRV-guided training approach has led to greater improvements in endurance performance when compared with “traditional” training programing (Kiviniemi et al., 2007, 2010; Stanley et al., 2013a). However, this training approach may not be as simple as previously thought. Under specific circumstances, such as following heavy training loads in the heat, increased, not decreased, vagal-related HRV indices have been observed within 24 h, despite an acute decrease in perceived wellness (Buchheit et al., 2013a). Additionally, during an intense multi-day desert race (i.e., Marathon des sables 2005, running 253 km in 7 days in extremely hot environment), after the expected initial drop in vagal-related indices during the first 3 days (Brenner et al., 1998), we observed a clear increase in cardiac parasympathetic activity, which did not match the reported increased perceived fatigue and drop in running performance (Figure 4). This inversed association between vagal-related HRV indices and acute fatigue suggests that in addition to the load of the preceding session(s), data on environmental conditions and hydration status need to be considered to correctly interpret the ANS response to exercise (Buchheit et al., 2013a; Stanley et al., 2013a). This is directly related to the fact that increases in plasma volume, that are usual responses to both intense aerobic-oriented exercise (Green et al., 1984) and heat acclimatization (Ladell, 1951), tend to increase beat-to-beat HRV, independently of clear changes in fatigue and/or fitness (Spinelli et al., 1999; Buchheit et al., 2009, 2013a). Finally, other factors influencing the recovery time course of cardiac autonomic activity, such as hydrotherapy and sleep should also be considered when interpreting daily changes in HRV (Al Haddad et al., 2012; Stanley et al., 2013b).

#### Chronic responses

**Figure 5:**
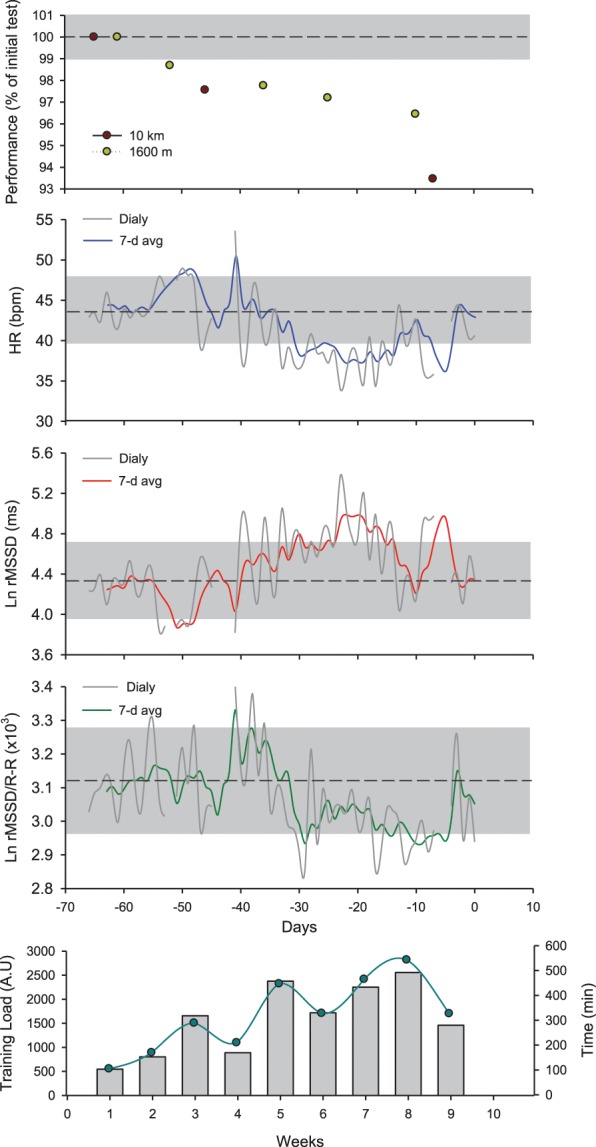
**Changes in running performance (best time over a self-paced 5 × 1600-m interval training session and an all-out 10-km run), resting heart rate (HR), logarithm of the square root of the mean of the sum of the squares of differences between adjacent normal R-R intervals measured at rest after awakening (Ln rMSSD), the Ln rMSSD to mean R-R interval (Ln rMSSD/R-R) and training load (perceived exertion, CR-10 Borg scale × training duration Impellizzeri et al., 2004) and volume in a distance runner (32 year-old, VO_2max_ = 59 ml/min/kg, vVO_2max_ = 18 km/h) over the 9-week training program leading to his first marathon (3 h 15 min).** The shaded areas represent trivial changes (see Table 2). Ln rMSSD and running performance change as a function of the training progression. While running performance improves continuously throughout, Ln rMSSD changes follow a bell-shaped relationship, with an initial increase followed by 2 consecutive reductions during the last phase of training. Interesting, while the decrease in Ln rMSSD during week 7–8 is concomitant with a plateau of the Ln rMSSD/R-R ratio, the decrease observed during week 9 occurs with an increase of the Ln rMSSD/R-R ratio. This suggests that the decrease during week 7–8 is likely related to a saturation phenomenon (further increase in vagal activity), while that seen at week 9 is more likely related to a decreased vagal activity/increased sympathetic activity (which might be needed to reach greater exercise intensity during competition). This interpretation has important implications when using HRV to guide the training process, and is only possible when using the combination of both indices.

It is now well established that during a training intervention over several weeks, HRV and HRR fluctuate in accordance to training load periodization (Pichot et al., 2000, 2002; Portier et al., 2001; Iellamo et al., 2002; Iwasaki et al., 2003; Garet et al., 2004; Manzi et al., 2009a; Buchheit et al., 2010a). When considering ANS activity over consecutive training blocks, moderate training loads are generally associated with increased vagal-related HRV indices, while high training loads, with decreased vagal-related HRV indices (Pichot et al., 2000, 2002) and slower HRR (Borresen and Lambert, 2007). However, in several studies in elite endurance athletes and/or athletes with long training history, the time course of cardiac parasympathetic activity throughout the training plan did not always follow these trends, and was in fact more bell-shaped (Iellamo et al., 2002; Iwasaki et al., 2003; Manzi et al., 2009a; Le Meur et al., 2013b; Plews et al., 2013b) (Figure 5). In these endurance athletes, cardiac autonomic regulation likely improves during the first part of the training phase (e.g., building-up or extensive endurance phase, likely leading to functional overreaching), while it decreases over the weeks preceding competition (i.e., tapering Hug et al., 2014). Since this particular pattern has been observed both in highly successful athletes (World and Olympic champions Iellamo et al., 2002; Plews et al., 2013b) and recreational athletes with long training history completing successfully their first marathons (Iwasaki et al., 2003; Manzi et al., 2009a; Hug et al., 2014), it may reflect an optimal training response. Importantly, performance keeps improving despite the decrease in cardiac parasympathetic activity (Figure 5), such that the relationship between these two variables becomes reversed during the final stages of preparation. In support of this, while there are generally positive correlations between vagal-related HRV indices and markers of aerobic performance when considering large groups of athletes of varying training status (Pichot et al., 2002; Buchheit and Gindre, 2006; Buchheit et al., 2010a), negative and large correlations have been reported in homogeneous groups of well-trained athletes (Bosquet et al., 2007; Buchheit et al., 2011a). The reasons behind this training phase- or load-specific relationships are not fully understood, but might be related to training intensity distribution during the different training phases (Stanley et al., 2013a; Plews et al., 2014a), independent of the actual level of fatigue. In fact, low-intensity exercise (which represents the greater proportion of training during high volume training) is likely to acutely increase vagal-related HRV indices within 24 h after exercise, while intense exercise (which represents the greater proportion of training during tapering) decreases those indices for 1–2 days (Stanley et al., 2013a). In elite rowers with no evident sign of accumulated fatigue, a large increase in time spent below the first ventilatory threshold was associated with a small increase in rMSSD, while a large increase in the time spend training at intensities above the second ventilatory threshold was associated with a small decrease in rMSSD (Plews et al., 2014a). Taken together, these data highlight the importance of considering training context (phases, training load and its distribution) when interpreting changes in HR-derived indices. Making inferences on the training status of an athlete based on HR measures, with no consideration for the training context, is likely misleading. However, it should be acknowledged that while assessing training load and training intensity distribution is straightforward in endurance sports (i.e., time between predefined intensity zones based on ventilatory break points, HR or blood lactate levels Plews et al., 2014a), this is more difficult in team sports, where the load arises from a myriads of biological systems stressed simultaneously. Therefore, whether the impact of training load (and intensity distribution) on HR-derived indices described in endurance sport (Plews et al., 2014a) is the same in team sports has still to be examined.

### Saturation of high-frequency oscillations of R-R intervals

The decrease in vagal-related HRV indices observed in athletes with long training history, in the absence of fatigue or overload, can be attributed to two different mechanisms. The first is a decreased parasympathetic activity in relation the training load distribution as discussed above, that can be reinforced by a possible pre-competition stress (Mateo et al., 2012; Morales et al., 2013). This increased sympathetic activity is likely beneficial for performance, since it may allow the attainment of greater exercise intensity during competitions (increased “maximal sympathetic mobilization” during high-intensity efforts Hedelin et al., 2001; Pagani and Lucini, 2009; Laursen, 2010; Parouty et al., 2010). The second is a saturation phenomenon (Kiviniemi et al., 2004), which occurs independently of any sign of fatigue and/or sympathetic overactivity (Buchheit et al., 2004, 2006; Kiviniemi et al., 2004). This reduction in vagal-related HRV indices is associated with low HR levels, and is the consequence of an increased, not decreased, vagal activity. The underlying mechanism is the likely saturation of acetylcholine receptors at the myocyte level: a heightened vagal tone may give rise to sustained parasympathetic control of the sinus node, which may eliminate respiratory heart modulation and reduce vagal-related HRV indices (Malik et al., 1993). Because vagal-related HRV indices more reflect the magnitude of modulation in parasympathetic outflow as opposed to an overall parasympathetic tone *per se* (Hedman et al., 1995), they decrease despite the increased vagal activity.

**Figure 6:**
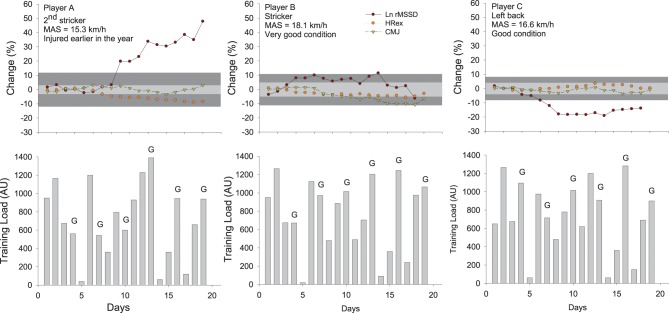
**Changes in the logarithm of the square root of the mean of the sum of the squares of differences between adjacent normal R-R intervals measured after exercise (Ln rMSSD), submaximal exercise heart rate (HRex), counter movement jump height (CMJ) and training load (session-rate of perceived exertion load × training/match duration Impellizzeri et al., 2004) in three highly-trained young soccer players during a competitive training camp.** The gray areas represent trivial changes (See Table 2) for HRex and CMJ (light gray) and HRV (dark gray). Errors bars (typical error of measurement, see Figure 7) have been omitted for clarity. While this might not be that clear when considering the actual spread of the TE, any change that is outside the gray areas is considered here as substantial (section Interpreting Changes in Heart Rate Measures: “Statistics are our weapons”). The changes in these variables in the three different players show different scenarios and illustrate how these indices can be used in combination to infer on training status and adaptations. Player A likely showed a positive adaptation to the camp (decrease in HRex and increase in Ln rMSSD), probably related to the fact that he arrived fresh and was a little bit detrained at the start following his previous injury. Playing at his position didn't require large high-intensity running demands (Buchheit et al., 2010c), so the balance between daily load and recovery was likely optimal for his fitness to improve (decrease in HRex) without compromising neuromuscular performance (CMJ remained stable). Player B, who was used to perform a very large amount of high-intensity actions during games as a striker, presented a stable fitness (HRex) and managed to maintain his ANS status into normal ranges, probably due to his very high fitness levels that may have allowed him to partially cope with the camp load (reduced relative intensity during games Mendez-Villanueva et al., 2013). However, neuromuscular fatigue progressively increased (decreased CMJ), consistent with the large playing demands. Finally, player C showed stable fitness and CMJ performance, but a clear decrease in HRV. He played the entire duration of games and in relation to his average fitness level, might not have completely coped with the load by the end of the camp. Nevertheless, the fact that his CMJ performance remained stable is consistent with the moderate neuromuscular demands of playing wide defender within his team's system of play (Buchheit et al., 2010c). MAS: maximal aerobic speed.

Since the saturation phenomenon can confound the interpretation of training-induced adaptations, several approaches have been developed to prevent its occurrence. This includes, in comparison with the usual supine recordings, the use of sitting (Kiviniemi et al., 2007), standing (Buchheit et al., 2010a; Schmitt et al., 2013) or post-exercise (Buchheit et al., 2008, 2010a) measures, where there is a minimal level of sustained sympathetic activity. This directly constrains vagal activity below the “tipping point” of saturation (i.e., R-R interval >1000 ms Kiviniemi et al., 2004; Plews et al., 2012, 2013b). When using resting supine or seated measures as recommended for convenience (section One Simple Variable, Multiple Complex Indices), the only way to know whether the decrease in the vagal-related HRV indices is related more to sympathetic overactivity vs. saturation, is to examine the changes in those indices (e.g., rMSSD) with regard to concomitant changes in resting HR. Normalizing HRV data for the prevailing R-R interval is now also used in clinical setting (Billman, 2013). In practice with athletes, we recommend computing the Ln rMSSD/R-R ratio (Plews et al., 2012, 2013b) (Figure 5; Table 2). In the case of a sympathetic-mediated decrease in Ln rMSSD, the R-R intervals will likely be shortened (higher HR), maintaining or eventually increasing the ratio. While moderate increases might be optimal (increased “maximal sympathetic mobilization”), extreme increases in the ratio might reflect maladaptation to training, and in turn, reduced performance (Plews et al., 2013b). In the case of saturation, the R-R is increased (lower HR), and the ratio is substantially reduced (Plews et al., 2013b). Whether saturation is beneficial for performance is difficult to decipher, because each athlete likely displays his own Ln rMSSD/R-R ratio profile (Plews et al., 2013a). The Ln rMSSD/R-R ratio profile is also training-cycle dependent, which suggests that longitudinal monitoring over months/years is required to optimize the overall monitoring process for each athlete (Plews et al., 2013a). For an athlete showing a saturated profile during extensive training periods, a sudden loss of saturation would suggest either an increased readiness to perform (positive adaptation Plews et al., 2013b) or the apparition of fatigue (negative adaptation Borresen and Lambert, 2008; Bosquet et al., 2008b). To decipher between these two scenarios, practitioners may consider the magnitude of the increase in the Ln rMSSD/R-R ratio (see above), and additionally use psychometric measures such as perceived wellness (McLean et al., 2010; Buchheit et al., 2013a), and/or monitor neuromuscular performance via counter movement jumps for example (Cormack et al., 2008; McLean et al., 2010) (Figure 6). Finally, recent results have also suggested that within-athlete correlation between vagal-related HRV indices and RR intervals may be stronger in over-trained endurance athletes compared with controls (Kiviniemi et al., 2013). Further longitudinal studies in larger groups of athletes are required to confirm the potential of this measure.

**Table 2:** **Guidelines for interpretation of chronic training status based on different scenarios in the changes in heart measures**.

			**Occurrence**	**Likely mechanisms**	**Practical interpretation**
**RESTING HRV AND HR**					
Changes in rMSSD	Changes in HR	Changes in rMSSD/RR ratio			
↑	↓	Maintained or moderate ↓	Very frequent following a short-term training program in moderately trained athletes, and/or during the building-up phase of elite athletes (high-volume and low-intensity training)	Increase in overall parasympathetic activity	Coping well with training
↑	↑	Large ↑	May occur at the beginning of a training block, likely observed in previously saturated athletes	Increased sympathetic activity which reverse the saturation phenomenon	If occurring short training blocks, increased readiness to perform If not, accumulated fatigue
↓	↑	Maintained or moderate ↑	Frequent during tapering	Increased sympathetic activity	If occurring during tapering, increased readiness to perform If not, accumulated fatigue
↓	↓	Large ↓	Frequent in elite athletes or others with a long training history	Increase in overall parasympathetic activity that causes saturation	Elite athlete/ athlete with long training history coping well with training, likely high-volume and low-intensity training If prolonged and not reversed with tapering, can inform on an overtraining state
**EXERCISE HR**					
↓			Frequent, in relation to changes in training load	Decrease in relative exercise intensity, plasma volume expansion	Cardiorespiratory fitness improvements
↑			Frequent, in relation to changes in training load	Likely increase in relative exercise intensity, or only reduction in plasma volume	Unclear; doesn't necessarily indicate decreased performance capacity
**POST EXERCISE HRR**					
↑			Very frequent following a short-term training program in moderately trained athletes, and/or during the building up phase of elite athletes (high-volume and low-intensity training)	Increase in overall parasympathetic activity	If occurring after a short training program, increased readiness to perform If prolonged and not reversed with tapering, can inform on an overtraining state
↓			Frequent during tapering	Increased sympathetic activity	If occurring during tapering after an overload period, increased readiness to perform If not, accumulated fatigue

Note that all changes need to be considered in relation to training context for appropriate interpretation (section Chronic Responses). HR, heart rate; HRV, HR variability; rMSSD, square root of the mean of the sum of the squares of differences between adjacent normal R-R intervals, reflecting cardiac parasympathetic activity; HRR, HR recovery.

Another promising approach is the use of the day-to-day variations in vagal-related HRV indices, which have been shown to decrease before non-functional overreaching (Plews et al., 2012). While still speculative, large day-to-day variations in HRV in response to daily changes in training loads might only be possible with a balanced and reversible ANS status, and is likely indicative of positive adaptation (Plews et al., 2012; Buchheit et al., 2013a). In contrast, in the case of a sympathetic over-activity, a day of rest/low training load may not be enough for parasympathetic activity to return back to baseline levels (Stanley et al., 2013a), which likely abolishes day-to-day variations in vagal-related indices (Plews et al., 2012). Similarly, i.e., the inability to reverse the ANS balance within 24 h, the consideration of day-to-day variations of vagal-related indices may also be of interest when the ANS balance is shifted toward a parasympathetic predominance (Hedelin et al., 2000). In this latter case, an exaggerated parasympathetic activity would maintain vagal-related HRV indices at high levels, even after some high-intensity sessions expected to acutely decrease those indices (Stanley et al., 2013a). Nevertheless, further research is warranted to confirm these latter hypotheses. It is worth noting that the consideration of within-athlete variation in HRV only makes sense when dealing with data collected at regular intervals (i.e., daily). The analysis of within-athlete variance using measures recorded at random intervals (Schmitt et al., 2013) is questionable and likely misleading.

## Interpreting changes in heart rate measures: “statistics are our weapons”

[Nick Broad, 1974–2013. Nick Broad was an English football nutritionist and sport scientist who worked for some of the biggest football clubs including Blackburn Rovers, Birmingham City, Chelsea Football Club and Paris St-Germain. Broad was a close friend of former Chelsea manager, Carlo Ancelotti. He graduated from Aberdeen University. Aged 38, he died on 19 January, 2013 of an accidental traffic collision (Wikipedia^1^)]. Nick was an example for a lot of sport scientists working in high performance, including myself. Among many others, I will miss our discussions on the monitoring process. This section is dedicated to him.

In sports, significant changes (i.e., based on a null-hypothesis testing approach) in either performance measures or physiological variables such as HR-derived indices may not be of practical significance, and conversely, non-significant changes can have meaningful effects (Hopkins, 2002). What actually matters to practitioners is whether the training-related change in HR measures could be important, i.e., whether their magnitude is actually greater than the smallest practical or meaningful change/effect (the so-called smallest worthwhile change, SWC) (Batterham and Hopkins, 2006; Hopkins et al., 2009). Additionally, in team sport athletes, comparing average changes in performance measures/physiological variables of different metrics and/or with heterogenous between-athletes variances can be misleading (Buchheit and Rabbani, 2013).

For groups of athletes, an attractive option to assess meaningful changes in a given variable and/or compare the changes in variables of different metrics is to use standardization (Hopkins et al., 2009). The observed changes can be standardized following Cohen's effect sizes principle (i.e., changes in the mean divided by the between-athlete SD of baseline test data), and their magnitude (in standardized unit) can then be compared with magnitude thresholds considered a small (>0.2–0.6), moderate (>0.6–1.2), large (>1.2–2) or very large (>2) (Hopkins et al., 2009). Because the SWC is generally a 0.2 standardized units, the assessment of substantial standardized changes is straightforward. However, because of the between-athlete variability in the responses [as evidenced by the confidence intervals (CI) for the standardized mean change], meaningful inferences about the observed magnitude have to be made to assess whether the effect is substantially beneficial/detrimental (clearly greater than the SWC), trivial (clearly within the SWC) or eventually unclear (CI overlaps zero and/or the SWC). These inferences can be achieved using a specifically-designed spreadsheet (i.e., post-only crossover for within-team changes Hopkins, 2006), where both the individual responses (CI around the average change) and the SWC and are taken into account to make the final interpretation (Hopkins et al., 2009).

**Figure 7:**
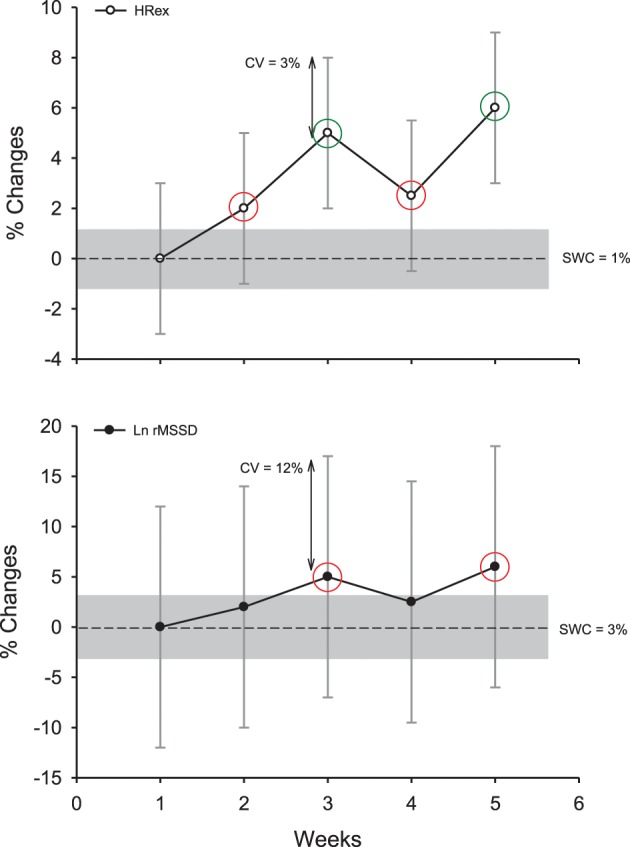
**Example of the decision process to interpret changes in exercise heart rate (HRex) and the logarithm of the square root of the mean of the sum of the squares of differences between adjacent normal R-R intervals (Ln rMSSD) in an individual athlete, while accounting for the uncertainty or the noise of each measure (expressed as a coefficient of variation, CV) and the so-called smallest worthwhile change (SWC; Table 2, where the gray areas represent trivial changes).** Green circles show clear and substantial changes; red circles show unclear changes although being out of the “trivial zone” (the CV overlaps both zero and the SWC). Note that despite similar percentage changes for the two HR-derived indexes, only changes in HRex are interpreted as substantial.

In practice however, practitioners need to monitor each athlete in isolation to individualize their training process, which requires a specific approach (Hopkins, 2004). As detailed in the following sections, the observed individual change in a given HR variable needs to be assessed in relation to the SWC (whether it is greater than the SWC, and if yes, how much), while considering the possible error of measurement (Figure 7). The following sections will detail how these two key elements can be determined.

### Acknowledging the uncertainty of the measures

When it comes to monitoring individual athletes, “normal” variations in the measures (e.g., within-athlete day-to-day variability, as measured by the typical error, TE, expressed as a coefficient of variation, CV) is the primary variable to consider (Hopkins, 2000a). The variability in each measure can be obtained either from the literature, or from repeated data collected in your own athletes (Hopkins, 2004). The magnitude of these variations depends both on the HR variable and the recording conditions (CV range: 3–190%, Table 1) (Sandercock et al., 2005). For example, the CVs for time-domain HRV indices at rest are generally lower than those of spectral indices (especially for the ratios, Table 1) (Al Haddad et al., 2011). Supine measures show lower CV than standing and exercise measures (Sandercock et al., 2005). The CV for HRV during exercise has been shown to be large, with some values ranging from 120 to 190% (Winsley et al., 2003; Leicht and Allen, 2008; McNarry and Lewis, 2012). The CVs for HRex or HRR measures (3–35%, Table 1) are clearly lower than those for exercise HRV (Bosquet et al., 2008a; Al Haddad et al., 2011; Dupuy et al., 2012), and their CVs tend to decrease slightly when exercise intensity increases (Lamberts et al., 2004, 2011a). However, because only submaximal exercises can be used as a warm-up in most of the sports, using a high-intensity exercise to derive less variable HRex and HRR data is not practical. When HR monitoring is used as a diagnostic measure, it is also unlikely that a coach would implement an intense exercise bout in athletes already suspected to be overreached.

Importantly, it is not the absolute TE (or CV) of a measure that matters, but the magnitude of this “noise” compared with (1) the usually observed changes (signal) and (2) the changes that may have a practical effect (SWC) (Hopkins, 2004) (Figure 7). A measure showing a large TE, but which responds largely to training can actually be more sensitive and useful than a measure with a low TE but poorly responsive to training. The greater the signal-to-noise ratio, the likely greater the sensitivity of the measure. To understand the possible sensitivity of the different HR measures to positive adaptations to training, training-induced changes in all HR variables (Buchheit et al., 2010a) were put in relation to the TE reported in the literature in a similar population (Al Haddad et al., 2011). The comparison of the signal-to-noise ratio calculated for each variable (range 0.7–1.6, Table 1) suggests that HRex might be the most sensitive measure to track positive aerobic-oriented training adaptations. However, these results might only apply in the context of the selected study (Buchheit et al., 2010a) (short training period aimed at improving aerobic-related performance). The sensitivity of HRex to performance improvements might decrease over longer training periods (Scharhag-Rosenberger et al., 2009), and whether it could be used to track other types of performance improvements (i.e., non-aerobically related) still needs to be examined. Less data exists on the sensitivity of HR measures to monitor negative responses to training, and importantly, there is no study where all HR variables have been reported simultaneously (which is required to compare the respective sensitivity of each HR index). In the recent study by Schmitt et al. (2013), the signal- (difference between fatigued and non-fatigued state) to-noise ratio was greater for HRV indices (i.e., 3 for a vagal-related index) than resting HR (1.3) in supine position. This difference in apparent sensitivity is consistent with the stronger trend toward non-functional overreaching for resting vagal-related HRV indices compared with resting HR (*r* = 0.88 vs. 0.81) reported by Plews et al. (2012). Considering that HRex may not be able to track impairment in physical performance (Buchheit et al., 2012), these data collectively suggest that resting HRV might be the HR measure that is the more sensitive to fatigue. Further studies are warranted to confirm this latter hypothesis.

The magnitude of the TE can also be reduced while repeating the measurements, since the noise decreases by a factor of 1/√n (i.e., using 4 tests halves the noise) (Hopkins, 2004). The interest of using repeated measures was recently shown in endurance athletes, where correlations with running performance in healthy (Plews et al., 2013a) and overreached (Le Meur et al., 2013b) athletes could only be observed using the average of at least 3–4 days of HR and HRV data, as opposed to isolated measures taken on single days (Plews et al., 2013a, 2014b). Nevertheless, we are all looking for a test with a TE that is lower or at least equal to the SWC, with the ideal scenario being a test with a TE that is less than half the SWC: any change in the measure that is greater than the SWC will be almost certainly substantial (Hopkins, 2004). For other scenarios, practitioners are advised to use common sense and consider (visually) how much the noise overlaps the SWC band (Figure 7). When extreme precision in the interpretation is required, a specifically designed spreadsheet (Assessing an individual, Hopkins, 2000b) can provide practitioners with the exact chances (%) for the observed change to be substantially greater or lower than the SWC.

### Determining the smallest worthwhile change

While defining the SWC is a key aspect of the monitoring process (Hopkins, 2004), it has often been overlooked in the literature (Borresen and Lambert, 2008; Daanen et al., 2012; Schmitt et al., 2013; Twist and Highton, 2013). A change that is clearly greater than the TE isn't obligatory meaningful practically: it has to be clearly greater than the so-called SWC to be meaningful. Defining the appropriate magnitude for the SWC is complex however, and likely depends on the training context, the type of adaptations which are aimed to be monitored, and the monitored variable itself. For individual sports performance, expressing changes as a fraction of the within-athlete variation in performance is probably the best standardization option, with the ability to compare the obtained scores with thresholds for magnitude. A third of within-athlete variation in performance between competitions (expressed as a CV) is generally considered as the SWC, while 0.9, 1.6, and 2.5 refer to moderate, large, and very large changes (Hopkins, 2004; Hopkins et al., 2009). Simulations have shown that such a 0.3 × *CV* performance improvement may give a top athlete one extra medal every ten races (Hopkins et al., 1999). However, when it comes to defining the SWC for individual changes in physiological markers such as HR variables (that are not actual performance measures) the appropriate magnitude for the SWC is less straightforward (Barnes et al., 2013). Within-athlete variations in HR variables have been used by some authors (i.e., SWC = 0.5 Le Meur et al., 2013b or 1 Plews et al., 2012, 2013b × *CV*), while others have used, as for performance variables in team sports, fractions of the between-athlete *SD* (Buchheit et al., 2010a, 2013a). Since the magnitude of the between-athlete *SD* is related to group heterogeneity and it doesn't consider the variations inherent to repeated measures, using the (individual) *CV* (Plews et al., 2012, 2013b) as the reference for the SWC is intuitively a better choice. However, there is currently no evidence that changes greater than any fraction of the *CV* would actually be meaningful in practice, with respect to actual changes in training status and/or performance. It is also worth noting that because of the training context-dependency of HRV changes (Plews et al., 2014a), the actual magnitude of the SWC may need to vary throughout the training phases. HRV responses to either isolated sessions (Stanley et al., 2013a) or prolonged training phases (Plews et al., 2013b) are also highly individual, so that the magnitude of the SWC for HR variables need to be individualized.

To provide a starting point toward the understanding of the magnitude of the SWC for each HR variable, group-average changes in HR variables following a successful endurance training period were extracted from the literature, and related to the associated changes in performance variables for running (Buchheit et al., 2008, 2010a, 2011b, 2012, 2013a) and cycling events (Lamberts et al., 2009; Mann et al., 2013)). The SWC for performance was considered as 1) 1% for peak incremental tests speed (Buchheit et al., 2010a, 2012) and 10-km running (Buchheit et al., 2010a) and 40-km cycling (Lamberts et al., 2009; Mann et al., 2013) time, and 2) 0.2 × between-athletes SD for Yo-Yo Intermittent Recovery (Buchheit et al., 2011b, 2013a) and repeated-sprint ability (Buchheit et al., 2008) tests. The HR-variable change corresponding to the performance SWC was then linearly extrapolated from the group-average Δperformance/ΔHR relationship in each study, and considered as the SWC for the HR variable of interest (Table 1). The SWCs obtained from each study was then averaged to produce single SWC estimate for each HR measure. This group-average approach was preferred to within-individual modeling for two main reasons: individual data points were not available for all studies, and the noise inherent to HR measures might compromise the performance/HR relationship at the individual level. Since the time course of HR may become bell-shaped when training is prolonged over several months, as reported in elite rowers (Iellamo et al., 2002; Plews et al., 2013a), triathletes (Plews et al., 2012; Le Meur et al., 2013b) or runners preparing for a marathon (Iwasaki et al., 2003; Manzi et al., 2009a and Figure 5), the use of the SWC provided in Table 1 might have to be restricted to moderately-long training periods (building-up phases), and/or performance improvements within the range of those observed in Buchheit's study (2010a). While this bell-shaped time course likely reflects an optimal scenario (rowers were gold medalist at the Olympic and all runners ran their personal bests), the fact that HR-variables returned to baseline levels by the end of the tapering phase, and the lack of information on performance changes throughout the training programs, do not allow a clear estimation of the SWC in these cases. However, it can be suggested that a ~4–9% increase in vagal-related HRV indices during the building-up phase may be required for the athletes to compete optimally following the taper (Iellamo et al., 2002; Plews et al., 2012, 2013a; Le Meur et al., 2013b) (Figure 5). Because of the limited data in the literature on negative responses to training, and the lack of consensus on the expected relationships between HR variables and poor training status, the magnitude of the SWC to assess negative adaptations has still to be investigated. Until new evidence is provided, I suggest using, for convenience, the same threshold as for positive adaptations.

### Making decisions

Guidelines to interpret the changes in the different HR measures are provided in Table 2. As emphasized in section Responses to Training Sessions, Training Intensity Distribution and Environmental Conditions, the training context is crucial to interpret these changes correctly. When monitoring athletes, there is always a risk for either false negative (no decision taken when you should) or positive (decision taken when you should not) errors. In elite sport, errors may have drastic consequences, therefore I recommend adopting a conservative approach to reduce false negatives (Buchheit et al., 2013c). It is probably better to “overcall” and reduce training load in athletes suspected of becoming tired/sick while they are not, than the conserve. In this sense, using a smaller SWC for negative adaptation might be an option. Once substantial impairment in the HR markers is identified, practitioners may have to decide whether they make decisions immediately (i.e., alter training contents with respect to the diagnostic), or wait for the changes to be observed 2–3 consecutive days/weeks (Brink et al., 2010). These rules of action can actually be gradual and/or index-dependent. For example, after a substantial decrease in resting rMSSD despite a maintenance of training load, the training intensity distribution of a few sessions could be shifted from intense to low (which is supposed to increase the rMSSD, section Responses to Training Sessions, Training Intensity Distribution and Environmental Conditions), without reducing the overall load. If this marker was to decrease further after the first training adjustments, the athlete could then be enforced to rest (diagnostic of fatigue) and/or to engage in a specific re-conditioning program (diagnostic of detraining, in conjunction with other markers, see below). Finally, further analyses with larger numbers of athletes showing clear changes in training status are still required to determine the exact sensitivity (the proportion of true cases that are diagnosed as cases) and specificity (proportion of true non-cases that are diagnosed as non-cases) of each HR measures.

## Ideal vs. real

If the objective is to guide daily training contents with HR-derived measures, the only appropriate option is to use resting (morning) HRV (Kiviniemi et al., 2007, 2010; Stanley et al., 2013a). The first advantage of morning measures is that there is time for the data to be analyzed prior to the training, that can then be modified accordingly. Secondly, resting HRV measures can be repeated at any time (Hautala et al., 2001) to assess the detailed recovery time course of the cardiac ANS following each training session/competition (Stanley et al., 2013a). In contrast, with exercise and post exercise measures, which are generally performed during the session warm-up, there is no time for analysis before training. Additionally, the frequency of data collection to track the ANS recovery time course is limited to the times when the athletes can exercise (e.g., no measure later in the day or during sleep Hautala et al., 2001; Stanley et al., 2013a).

When it comes to the selection of the most appropriate measure for moderate-to-long term training monitoring with athletes throughout the season, practitioners must consider the balance between the sensibility and power of a given measure (e.g., its relevance in relation changes in fitness, fatigue or both, its sensitivity to training Buchheit and Rabbani, 2013), and the practical possibilities of implementation (e.g., daily vs. weekly measures). This directly determines the overall usefulness of the “monitoring system.” Considerating the needs and the time constrains of each athlete, a measure that can be collected at a high resolution (i.e., more frequently) is likely more useful than a possibly more powerful measure, but that can only be collected occasionally. Increasing measurement frequency allows defining more accurately the normal ranges of individual variations and decreases the noise of measurement by a factor of $1/\sqrt{n}$, which, in turn, improves our ability to adjust training contents. The perfect scenario would be to collect a combination of the most powerful measures on daily basis.

**Figure 8:**
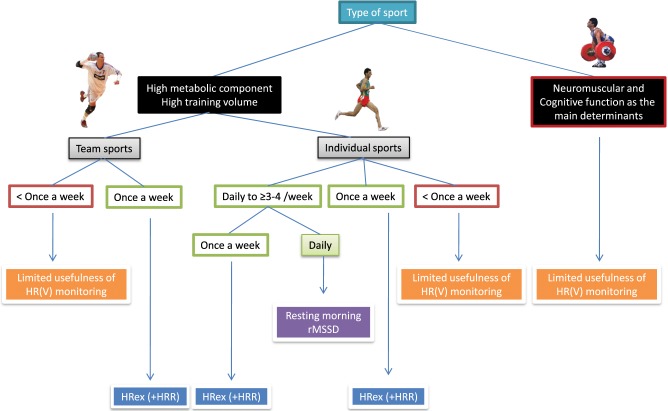
**Decision chart for the selection of heart rate (HR) measures based on sport participation and implementation possibilities.** HRV, heart rate variability; rMSSD, logarithm of the square root of the mean of the sum of the squares of differences between adjacent normal R-R intervals measured after exercise; HRex, submaximal exercise heart rate; HRR, heart rate recovery.

Figure 8 shows a simple decision charts that may help practitioners to select the most appropriate HR measure(s) for their monitoring system, based on their type of sport and the possible timing of each measurement. The minimum number of daily recordings per week required to assess meaningful changes in training status from HRV is an important consideration with respect to athlete's compliance. Therefore, endurance athletes might be advised to collect HRV upon awakening at least 3–4 times a week (Plews et al., 2014b) (for the data to be averaged Le Meur et al., 2013b; Plews et al., 2013a,b), and supplement these measures once a week with HRex (and HRR). These athletes are typically at a greater risk of overtraining than team sport players, therefore requiring, a more complete assessment. This population is also generally well receptive to frequent HR monitoring and can cope with (almost) daily recordings. By contrast, implementing HR measures more frequently than once a week is unrealistic in team sports, and home-based measures are idealistic; the only viable option is then to collect HRex (and eventually HRR) each week on a standardized training day. Furthermore, the measure of HRex can be integrated into the team warm-up, fitting well with the time requirement of elite team sports.

## Limitations of HR-measures

**Figure 9:**
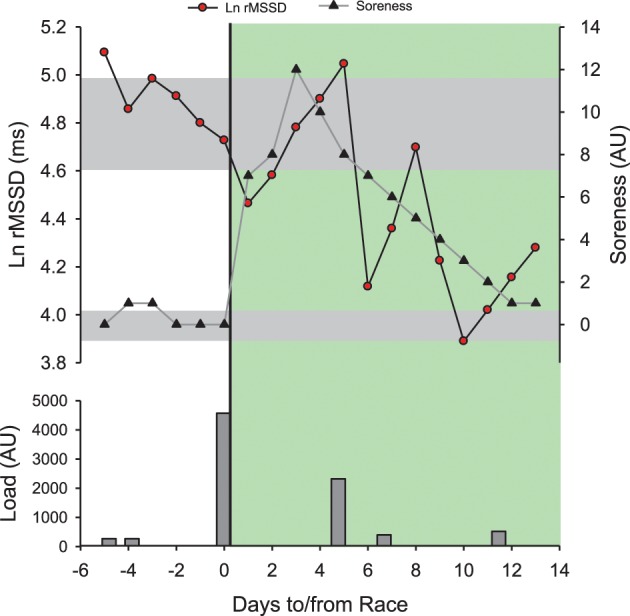
**Time course of the logarithm of the square root of the mean of the sum of the squares of differences between adjacent normal R-R intervals measured supine at rest in the morning (Ln rMSSD) and perceived muscle soreness (Soreness, 0–10 scale) in a distance runner (32 year-old, VO_2max_ = 59 ml/min/kg, vVO_2max_ = 18 km/h) before and after his first mountain trail (53 km, 4400 m of positive ascent in 8 h 22 min).** Training/competitive load was provided as perceived exertion (CR-10 Borg scale) × training/race duration (Impellizzeri et al., 2004). All sessions were run-based except the one at post-race +5 days which was a bike session. The green area represents the post-race recovery period. Gray areas represent trivial changes for both Ln RMSSD and Soreness. Note that post-race Ln rMSSD recovers within 2 days and rebounds above pre-race levels within 5 days. The recovery time course of Ln rMSSD unexpectedly mirrors the dramatic increase in post-race Soreness. The impressive increase in Soreness (12/10 scale!) is likely related to the fact that the runner was only used to run on flat courses (i.e., marathon training, Figure 5) and not specially prepared for mountain running at that time. The post-race changes in HRV follow initially the expected hemostasis recovery (rebound), and then likely reflect a detraining state after the first week of inactivity.

It is important to mention however that HR-measures cannot inform on all aspects of wellness, fatigue and performance (Buchheit and Laursen, 2013; Stanley et al., 2013a). For example, while results from a recent study suggest that cardiac parasympathetic recovery may recover in parallel with muscular performance (Chen et al., 2011), there was no association between the recovery of cardiac ANS and blood creatine kinase or perceived muscle soreness following both a soccer game (Buchheit et al., 2011b) and a 53-km mountain-trail run (Figure 9). Similarly, one could wonder how cardiac ANS could track metabolic recovery such as glycogen resynthesis (Buchheit and Laursen, 2013; Stanley et al., 2013a). Finally, the time course of HRex and HRV adaptation during a training camp at 3600 m in highly-trained young soccer players were dissociated from both the changes in perceived exertion response to a submaximal run and psychometric measures of altitude tolerance (Buchheit et al., 2013b). While the mechanisms remain to be elucidated, the lack of sensitivity of HR measures to some neuromuscular, metabolic or psychometric perturbations may be related to the fact that HRV is only a marker of *cardiac* ANS (Task Force, 1996). Therefore, the use of HR measures in combination with daily training logs and other non-invasive markers such as countermovement jump (neuromuscular fatigue Cormack et al., 2008; Hamilton, 2009; Buchheit et al., 2010b) and psychometric questionnaires (perceived general wellness and fatigue Hooper and Mackinnon, 1995) may offer a complete solution to monitor training status in athletes (McLean et al., 2010; Buchheit et al., 2013a). Therefore, assessment of the changes in all markers is required to make the right decision (section Making Decisions). The sport scientist then must deciding which variable/physiological system requires attention. As shown in Figure 6, during congested periods of play in young soccer players, different scenarios of adaptations may occur for different athletes: fatigue can occur at the neuromuscular level without an impaired cardiorespiratory fitness, and the converse. A last limitation of HR measurements is that athletes are generally required to use a chest belt, which is not always convenient. There is however no doubt that future technological development allowing the collection of heart beats via other types of sensors (e.g., wrist-only monitors, clothes, sheets) will further increase the interest of HR monitoring in athletes.

## Conclusion

To conclude, from a measure as simple as HR, a large number of indices can be computed, depending on the recording conditions and the associated signal analysis. The data reviewed in the present manuscript suggest that 5 min of resting HR(V) (Plews et al., 2013b; Stanley et al., 2013a) aimed at capturing cardiac parasympathetic activity, together with submaximal exercise HR (last min of a 4–5 min cycling/running bout) are likely the most useful monitoring variables. Resting HRV measures can be collected very frequently to both examine acute (daily recordings Stanley et al., 2013a) and chronic (at least 3–4 days per week Plews et al., 2014b) responses to training, and are likely sensitive to both positive and negative adaptations. Exercise HR may provide information more exclusively on chronic and positive adaptations. While HRR has been suggested to be of similar usefulness (Daanen et al., 2012) and sensitivity to fatigue and performance changes (Table 1), recent data in team sport athletes indicates otherwise (Buchheit et al., 2012, 2013a). Further, recording HRR requires a few extra minutes, therefore, the exclusive use of HRex may be most appropriate within the busy schedule of team sport players.

Correct interpretation requires each variation to be considered (1) in relation to the actual training phase and/or load the athlete is involved in, and (2) while considering both the individual typical error of measurement and the so-called smallest worthwhile change. In practice, the final decision to use a given HR measure should also be based on the level of information that is required by the athletes, and the possible frequency of measurements—both of which are likely sport and athlete-dependent. It is also important to consider that a measure that can be collected at a high resolution is likely more useful than a possibly more powerful measure that can only be collected occasionally. Finally, since HR-measures cannot inform on all aspect of wellness, fatigue and performance (Buchheit and Laursen, 2013; Stanley et al., 2013a), their use in combination with other psychometric and non-invasive performance markers may offer a complete solution to monitor responses to training in athletes (McLean et al., 2010; Buchheit et al., 2013a).

## Author contributions

Martin Buchheit had the original idea of the paper, wrote the paper and prepared the figures.

### Conflict of interest statement

The author declares that the research was conducted in the absence of any commercial or financial relationships that could be construed as a potential conflict of interest.

## Footnotes

^1^Wikipedia. http://en.wikipedia.org/wiki/Nick_Broad [Online]. (Accessed 20 Nov 2013].
